# From Passive Overeating to “Food Addiction”: A Spectrum of Compulsion and Severity

**DOI:** 10.1155/2013/435027

**Published:** 2013-05-15

**Authors:** Caroline Davis

**Affiliations:** Kinesiology & Health Sciences, Faculty of Health, York University, 343 Bethune College, 4700 Keele Street, Toronto, ON, Canada M3J 1P3

## Abstract

A psychobiological dimension of eating behaviour is proposed, which is anchored at the low end by energy intake that is relatively well matched to energy output and is reflected by a stable body mass index (BMI) in the healthy range. Further along the continuum are increasing degrees of overeating (and BMI) characterized by more severe and more compulsive ingestive behaviours. In light of the many similarities between chronic binge eating and drug abuse, several authorities have adopted the perspective that an apparent dependence on highly palatable food—accompanied by emotional and social distress—can be best conceptualized as an addiction disorder. Therefore, this review also considers the overlapping symptoms and characteristics of binge eating disorder (BED) and models of food addiction, both in preclinical animal studies and in human research. It also presents this work in the context of the modern and “toxic” food environment and therein the ubiquitous triggers for over-consumption. We complete the review by providing evidence that what we have come to call “food addiction” may simply be a more acute and pathologically dense form of BED.

## 1. Introduction

By many accounts, obesity now ranks as one of the greatest and most preventable global threats to public health [1, 2]. Furthermore, as a result of fetal programming, it will likely gather momentum and become a self-perpetuating problem as a form of “inheritance of acquired characteristics” from one generation to the next [3–5]. Currently, more than half the adult population is described as overweight or obese, (Implied in the terms overweight and obese is an excess in one's level of adiposity or percentage of body fat, not in other components of the mass that comprises their body weight such as muscle or bone density. In other words, body weight (typically expressed as body mass index (BMI): (weight kg/height metres2)) is typically and conveniently used as a surrogate for adiposity—although as an estimate of fatness, it has a considerable margin of error at the individual level.) marking the first time in human evolutionary history that the number of people with excess body weight has surpassed those who are underweight [6, 7]. Indeed, in the United States alone, as many as one in three adult women now have a BMI greater than 30 [8]. Moreover, and on a similar trajectory, estimates of the global prevalence of this condition in children increased from 42% in 1990 to 67% in 2010 [9]. During the same time period, both mean maternal weight at delivery and mean offspring birth weight were steadily increasing [10]. These are important issues since maternal obesity is a strong predictor of fetal insulin resistance [11] and childhood obesity [12, 13]. 

Reflecting the obesity “pandemic”—with its prodigious economic burden on societies worldwide [14]—has been the relatively sudden and very pronounced global increase in obesity-related research since the end of the 20th century [15]. While several factors coalesced to serve as catalysts, what largely prompted this research focus was the powerful evidence, which had steadily accumulated since the 1930s, of a broad range of health risks associated with possessing too much fatness, including links to metabolic, cardiovascular, and oncological disease, as well as to orthopedic complications and psychiatric disturbances [16, 17]. 

At the beginning of the 21st century, there was a noticeable migration from the earlier research emphasis on health-related* outcomes* of obesity to an emerging focus on the *causes *of, and *antecedent factors* leading to, obesity. This new endeavor was prompted, in part, by several important scientific developments such as the discovery of leptin [18], technological advances in genetic research including the sequencing the human genome [19], and the burgeoning field of neuroscience with its sophisticated neuroimaging techniques [20]. At about the same time, political influences also played an important role in bringing about a new direction in research. Public health officials and advocates began a relentless exposition of the dramatically changed food environment and of the food and beverage industry's powerful role in promoting poor nutrition policies [21–23]. 

Some of the ‘highlight' observations and conclusions from the resulting body of research were that most animals, including *homo sapiens*, are biologically predisposed to overeat when energy resources are available and that we do this with alacrity when foods are of a particularly appealing composition [24, 25]. This once adaptive biological drive is now contributing to widespread overnutrition in a fundamental way, especially because economic growth in capitalist cultures began to switch from a demand for cheap labour to an emerging emphasis on consumption and how best to drive consumer behavior [5].

### 1.1. Evolution and Energy Resources

These events raise the interesting and topical question: *why* are individuals prone to eat, often voraciously, beyond caloric need and to store an excess of adipose tissue when the long-term consequences of these inherent strategies are so remarkably deleterious? A biocultural viewpoint, highlighting co-evolutionary processes, is, undoubtedly, the best viewpoint for understanding these issues [26]. Since such a rapid and recent change in the population phenotype could not be genetic, one must assume that our current environment interacts with our evolved biology, in some complex way, to give rise to the situation where a substantial proportion of the population are prone to persistent weight gain [4].

Evolutionary theory pivots on the fundamental premise that most characteristics we possess resulted largely from natural selection such that gene frequencies of alleles increase across generations because they confer reproductive success, that is, more progeny are produced who survive until they are procreant. And energy availability is the most important environmental factor for sustainable reproduction [27]. The crux of the issue, however, is that in most environments, and for most species, energy availability has been historically inconsistent and unpredictable. In other words, it has typically been difficult to obtain even ample resources let alone a surplus of food—at least until modern times during which there have been dramatic changes to the food environment. In many parts of the world, the industrialization of food processing and the advanced technology to enlarge food supplies have created an ubiquitous, greatly available, and inexpensive source of energy-dense (high sugar and fats) food, and concomitantly, pronounced reductions in the need for energy expenditure to secure this food [26]. 

 Another basic tenet of evolutionary biology is that even though a gene ultimately decreases the lifespan, if it increases the ability to produce offspring and raise them to maturity, that gene will be selected. Schneider [28] argues that, historically, the mechanisms that prioritized eating or copulating were very successful at perpetuating our species. In other words, the tendency to engage in these behaviours evolved in an environment where there was typically a choice between time spent foraging and feeding, and time spent courting and mating. A compelling body of recent and related animal research has demonstrated that leptin increases sexual motivation in females independent of the effects of the estrous cycle [29], and that in males it also increases the relative motivation to engage in sexual activity rather than in eating behaviours [30]. In modern times, these same mechanisms have likely contributed to increases in obesity because leptin levels increase as weight is gained. Specifically, obesity is a highly transmissible condition (~.6 to .8) suggesting a mainly genetic influence on population variation in BMI [31, 32]. Heritability estimates for obesity are also greater in women than in men [33, 34]. Since obese mothers tend to have significantly more children than their lean counterparts, obesity is therefore likely to increase from generation to generation for purely genetic reasons [35]. 

### 1.2. Overconsumption and Weight Gain

While the population prevalence of obesity has doubled in the past few decades, its morbid form (BMI > 40) has seen an alarming 4-fold increase compared to cohorts with lower BMI values [36]. These findings suggest that, on average, the more weight one gains, the easier or more likely it is that the process will escalate over time. The issue of “overeating sensitization” will be discussed in greater detail in a later section of this paper.

At a basic physiological level, the only way to accumulate body fat is by a sustained state of positive energy balance. A question that continues to be debated, however, amongst scientists trying to explain the rise in obesity rates is the relative contribution of chronic increases in energy intake on the one hand, as against chronic decreases in energy expenditure on the other [37]. In a sense, this is a question without a clear answer because, in the context of energy balance, the two contributing factors are rather difficult to uncouple. Nevertheless, some related evidence seems rather indisputable. For instance, data from a variety of sources have consistently demonstrated an increasing trend in daily calorie intake in most developed countries over the past few generations [14, 38]. Also, in most high-income countries, energy expenditure has been decreasing since the beginning of the 20th century—to the very low levels we see today—largely because of mechanization, urbanization, and computerization [14]. 

In a well-modulated biological system we would expect decreases in physical activity to be matched by decreases in food intake, as was generally seen in the first half of the 20th century. Swinburn et al. [14] have described the “energy balance flipping point” (pg 807), a phenomenon which occurred around 1960, when population energy intake no longer matched output. This, he argued, occurred almost entirely because of the emergence, and great availability, of highly caloric and very tasty processed foods whose characteristics disrupted the homeostatic mechanisms that had evolved to keep intake and expenditure roughly in balance, except, of course, in times of unavoidable energy scarcity such as seasonal food shortages and periods of drought or botanical pestilence. 

This singular shift in the direction of weight gain in the past several decades inspired a novel research focus on the issue of *nonhomeostatic* drivers of food intake. There was a pressing need to better understand the universal human tendency to eat beyond caloric need—and in the absence of physiological signals of hunger—when individuals are in the presence of a rich variety of highly palatable and visually attractive food. Descriptively, the term “hedonic hunger” has been used to depict the imaginative cravings associated with these foods, and the motivational drive which fosters eating primarily because of the gustatory rewarding properties of the cuisine [39]. In an environment where hyperpalatable foods are abundant and proliferate, hedonic hunger contributes in a powerful way to patterns of overeating such as frequent snacking [40] and larger portion sizes [41], and the consequential weight gain that can lead to obesity. 

While the physiological mechanisms regulating homeostatic hunger are generally well understood [42–44], a clear understanding of the neural and peripheral brain pathways driving hedonically-based eating is only beginning to emerge. For example, it is now recognized that highly palatable foods activate the mesocorticolimbic reward circuits of the brain by the release of dopamine, as well as other neurotransmitters and hormones like the endogenous opiates [45]. Indeed, after a period of food deprivation any wholesome food will activate the reward pathway, which evolved to regulate the experience of pleasure from behaviours that are essential for our survival, and to enhance the motivation to seek out these rewards in our environment. The important distinction, however, is that delicious foods activate the neurochemical signals more reliably and more potently than less tasty substances. 

Recent experimental findings also indicated that when the motivation to eat is activated by highly palatable food such as pastries and cakes, and there is no prior and extended period of energy deprivation, activation of two other rewarding chemical signals—endocannabinoids and ghrelin—occurs [46]. Importantly, this hedonic response was not generated when bland foods like dry bread and milk were offered to participants under the same laboratory conditions.

## 2. The Modern Food Environment

 The dramatic rise in obesity rates in the past few generations can mostly be explained by pronounced changes in the physical environment and in people's personal economic environment [47]. In particular, a reduction in the time-cost of food across the global food system has been a major contributor to recent population weight gain [14]. What has been described as a markedly “toxic” food environment is also exemplified by the increased prevalence of chemical additives and flavor enhancers in the food supply [48]. Some have even suggested that the highly processed foods we eat would be more correctly labeled “food-like products” to distinguish them from the natural sources of energy like fruits and vegetables that comprised the diet of our ancestral predecessors [49].

The marketing power of palatable foods is not only seen by the high consumer demand for these products and the double-digit growth of goods like luxury chocolate, but by the invocation of food images to sell a host of other nonedible products. For example, the odorants currently used to sell bath and beauty products, and perfumes and colognes, now regularly imitate the scents and aromas of tasty and beloved foods, rather than the floral and botanical alternatives like lavender and gardenia that were traditionally used. A poignant illustration is the spring 2013 Special Promotion, by a very successful fragrance manufacturer, of their *Sugar and Spice* collection inspired by “delectable ingredients” that are “decadently tempting.” Their items bear names such as “Lemon Tart,” “Bitter Orange and Chocolate,” and “Ginger Biscuit.” 

Given the profound alterations to modern life on many parts of the globe, there has been much speculation about a current “mismatch” between our evolved human propensity to consume good tasting food when it is available—even in the absence of hunger—and the much-altered food environment where high-energy resources are prodigiously and excessively available [50–53]. Research has also focused on the complex interaction between the “human epigenome” and the modern food supply. For instance, there is accumulating evidence that maternal body weight and diet may work, via *in utero* placental signaling, to alter her offspring's future eating behavior and risk for obesity [4]. 

Globally, the prevalence of obesity is greater in women than men [54]; similar data were also found in those of African descent and in Hispanics in the United States, but for some reason, not among American Caucasians [8]. In a recent study, and using as evidence 20th century famines that occurred around the world, it was shown that the female offspring of women whose diets were nutritionally restricted during their pregnancy, and who later found themselves raised in an environment with an abundance of calorie-rich food, were at greater risk of obesity than their male counterparts [55]. Interestingly, this phenomenon is most visible in moderately developed countries where the economy has expended rapidly in recent years, illustrating the strength of the *in utero* or perinatal nutritional environment in altering risk for obesity. These findings also provide some insight into factors contributing to global sex differences in obesity prevalence. Since the modern food environment also increases the likelihood that some individuals will develop a compulsive pattern of overeating and show signs of dependence on sugary and fatty substances (to be discussed later in this review), it may be that women are also more prone to “food addiction” than men.

### 2.1. Brain Reward Mechanisms and Food Consumption

For at least half a century, we have known that dopamine neural circuitry in the midbrain modulates the reinforcing properties of addictive drugs like alcohol, psychomotor stimulants (“speed”), and nicotine [56]. Surprisingly, however, the regulatory role of these same pathways in relation to food consumption—especially when the diet is highly palatable—has only been investigated systematically for a few decades [57]. Somehow, the chronology of these respective scientific “discoveries” seems rather upturned—like the proverbial “cart before the horse.” In other words, the time sequence implies that the human brain somehow evolved to find drugs of abuse highly reinforcing, and that coincidentally, these same brain regions are activated, albeit less potently (It has been estimated, for instance, that the dopamine release from psychomotor stimulant drugs is about 10-fold greater than to a normal meal when one is hungry [58].), by a wide variety of tasty foods. 

Anthropological evidence indicates that human beings shared a co-evolutionary relationship with psychotropic plant substances, such as the coca leaf and the poppy, in prehistory for millennia. Indeed, they frequently ate them as food because their ingestion solved a recurrent problem faced by our ancestors [59]. Due to famines and seasonal food shortages, people experienced deficits in the brain's “chemical messengers” (e.g., dopamine and serotonin) with considerable regularity—depletions which can adversely affect critical behaviours and emotions like motor activities, cognitive abilities, and mood [60]. Because these plant-based “neurotransmitter analogues” prevented fatigue, diminished appetite, and increased tolerance for hunger when they were eaten, they helped to counteract the deleterious consequences of stress if adequate food sources were challenged [59]. In other words, plant substances served the dual purpose of substituting for more costly energy and of buffering against the biological ravages of prolonged strain and tension. 

It is well established that the mesocorticolimbic dopamine system, and its neurophysiological connections, evolved to subserve primitive and necessary biological functions long before the concept of addiction was relevant and even considered [61]. These neural pathways originate in the ventral tegmental area, which sends projections to the limbic structures, including the amygdala, and to the striatal regions of the basal ganglia. There are also afferent and efferent connections between these subcortical structures and parts of the prefrontal cortex [62]. Collectively this brain circuitry is also known as the *common reward pathway* because we experience pleasure and an enhanced sense of well-being from its activation, which occurs when we engage in a wide variety of activities, like eating, sex, and maternal behaviours, that are essential for our continued existence. More broadly, these midbrain pathways comprise both a survival and a reproductive regulatory system because they respond to perceived threats against our reproductive fitness, as well as to the positive behaviours we find rewarding [63]. 

Substance abuse is a relatively recent phenomenon in the history of our species and occurs largely because we learned to purify and synthesize potent drugs from plant materials and because their routes of administration to the brain have become more direct [64, 65]. In other words, it is the concentrated doses of these substances, and their ubiquity, that have contributed to wide-spread human drug addictions. Consequently, it seems reasonable to conclude that organic compounds in our environment—whether they derive from psychotropic plant substances or from edible energy resources—can only be viewed as *beneficial* or *harmful* when we take account of their potency, the frequency of their consumption, and the relevant characteristics of those who partake of them [66].

### 2.2. Some Foods as Addictive Substances

Of all the macronutrients, carbohydrates—and in particular disaccharides like table sugar (sucrose)—have been ascribed the greatest, potentially addictive, properties [67]. It is hardly surprising, therefore, that sweet-tasting foods are the ones we most prefer to eat, and that this preference is especially enhanced when foods contain fat and salt in addition to sugar. It appears, however, that in the quantities we currently consume these highly palatable ingredients, they have a capacity for abuse similar to other addictive drugs [67–71]. The poignant observation that most rats show a stronger preference for a sweet solution than for cocaine—even in animals with an extensive history of drug use—is a strong testament to the powerful effects of sugar and its great reinforcing value [72]. 

As we have seen, a substance's addictive potential is increased both by its potency and by its rapid absorption into the bloodstream [71, 73]. Relevantly, sugar is quickly absorbed and thereby substantially more reinforcing than many other foods. As foods became more processed and altered by the manufacturing industry, they began to take on more and more properties of addictive drugs, largely because of the ingredients that were added to enhance the taste of food [70]. A particularly powerful example is the evolution of corn in the human diet. For generations, it was a staple and frequently consumed source of complex carbohydrate. Due, however, to advanced technological and subsidies to the corn farmers in the United States in the 1960s and 1970s, this crop was refined into high fructose corn syrup (HFCS), an exceedingly concentrated and very sweet simple carbohydrate. Otherwise fructose is found only in small amounts and in a few of the natural foods that comprised most traditional diets [74]. Over the past 40 years or so, the abundance of HFCS—added as a sweetener to a large variety of processed foods such as soft drinks, breakfast cereals, and desserts—has been a powerful contributor to the greatly enhanced palatability of many of the foods we eat regularly. Indeed, our consumption of this inexpensive simple sugar has increased substantially in a few generations from about 4% to 12% of our daily caloric intake [75]. 

The special physiological properties of fructose render it very similar to substances like alcohol because the two are biochemically congruent; ethanol is simply the fermented byproduct of fructose [76]. When taken in large quantities, both substances can promote neurobiological changes that encourage further problematic use such as excessive overconsumption. As with all addictive drugs, excessive consumption causes a dampening of the brain's “pleasure” signal, which can foster increased intake of the substance, pronounced cravings, continued use despite negative consequences, and withdrawal symptoms during periods of abstinence [77]. 

The metabolic impact of fructose also differs substantially from other sugars like glucose because it blunts leptin signaling, thereby promoting sensations of hunger and activation of the reward pathways creating a desire for consumption independent of energy needs [78]—a phenomenon that is directly comparable to what occurs when alcohol is used [76]. In other words, while glucose stimulates the release of insulin, decreasing the desire to eat, fructose and ethanol produce this effect to a very weak degree. Interestingly, etiologic links between overeating and alcohol use have also been demonstrated in a recent study. Women with a family history of alcoholism (i.e., a biological parent or sibling) had almost double the odds of being obese compared to those without a family history—a statistical association that remained robust after controlling for a number of well-established covariates of obesity [79]. While this relationship was seen in men as well, it was not as strong as in women.

Diets high in concentrated fats and sugars are not only dense in calories, but metabolically efficient because they provide a large proportion of daily energy requirements. They also tend to elevate mood by releasing neuropeptides which reinforce their selective preference [80]. Although hyperpalatable foods differ in some ways from the key characteristics of traditional addictive drugs, they also have many features in common [70]. For instance, highly processed foods and drugs of abuse are both capable of triggering cravings [81, 82]. Biologists believe that “cravings” for sugar and fat evolved to enhance human energy intake in unpredictable nutritional environments [66]. Consumption of highly processed foods and drugs of abuse are also both associated with compulsive overuse even in the face of severely adverse consequences [83]. And finally, the loss-of-control over intake and the inability to cut down consumption of both substances are additional characteristics they have in common [83]. In summary, while highly processed foods do not cause the intoxication of alcohol or the euphoria produced by some stimulant drugs like cocaine, there are nevertheless significant areas of overlap with conventional addictive drugs [70]. 

### 2.3. Compulsive Overeating as an Addictive Behaviour

The parallels between compulsive overeating and drug abuse have been recognized by clinicians for decades, albeit mostly in anecdotal reports, and with particular reference to the pronounced cravings and the mood-enhancing effects they produce [70, 84–90]. Among the general public, the notion that overeating can be an addictive behavior has also been widely accepted for many years—as seen, for instance, by the many 12-Step treatment programs (e.g., *Overeaters Anonymous* founded in 1960; *Food Addicts Anonymous *founded in 1987) established for those who suffer from an apparent loss-of-control over food intake [91]. Interestingly, however, a recent study, using both surveys and an experimental paradigm, found that the public perception of “food addiction” was more favourable than it was for other addictions like drug and nicotine abuse, and alcoholism [92]. Consequently, this putative condition may be less vulnerable than other addiction disorders to the ill effects of social stigma.

Although there are no formal or agreed-upon diagnostic criteria, the notion of an addiction to hyperpalatable food is also receiving widespread and increased attention from the popular media. It is only very recently, however, that the term “food addiction” has gained any scientific credibility. For instance, according to the Web of Science data base, there has been a steeply exponential increase in the number of scientific publications devoted to this topic, from 3 in 2008 to 34 in 2012, and only two publications between 2000 and 2007. An important catalyst for the renewed interest in food addiction is the very convincing and accumulating evidence—illustrated in the previous section—that highly palatable foods rich in sugar, fat, and salt have an abuse potential similar to addictive drugs like cocaine and alcohol [67, 70, 71]. In addition, evidence of the biological parallels between drug and food abuse, as demonstrated by preclinical experiments and behavior genetic research, has further strengthened the credibility of the food-addiction construct [93, 94]. Functional neuroimaging studies have also demonstrated that pleasant looking, smelling, and tasting foods have reinforcing properties similar to drugs of abuse [95]. 

 Drug addiction is a dynamic behavioural disorder progressing from intermittent to regular use, and eventually to excessive and intractable intake. Evidence from population-based transmission studies also suggests that *common* biological influences contribute to the abuse of a broad range of addictive substances and that these factors are of equal importance in men and women [96]. While natural and pharmacologic reinforcers activate both limbic and cortical structures and connections known collectively as the mesocorticolimbic dopamine pathway (described in a previous section) [97], the pleasure experienced from either tasty foods or addictive drugs correlates positively with the amount of dopamine released specifically in the striatum [98, 99]. Not surprisingly, individual differences affecting the responsiveness of the dopamine system have been a major target for investigating vulnerability to drug abuse [100] and more recently, risk for compulsive overeating [94, 101, 102]. 

One source of evidence for a common biological connection between overeating and drug taking comes from a converging body of human and animal research demonstrating that excessive food consumption and drug self-administration are closely related genetically [103, 104]. For example, in a study of female twins, there was a moderate to large degree of overlap in additive genetic factors between alcohol use and binge eating, and no overlap in environmental effects [105]. Rats selectively bred for high sweet preference also represent a robust (drug) addiction-prone phenotype compared to the low sweet-preference group who appear to be an addiction-resistant animal model [103]. Similarly the inverse also obtains. Sugar preference is also greater in animals with high alcohol responsiveness compared to their low-responsive counterparts [106].

#### 2.3.1. The Development of Tolerance

Regulatory or homeostatic systems evolved to respond to challenges to the normal “evolutionary experience” of the organism and to make suitable compensatory adjustments [4]. When any homeostatic system remains chronically activated, or activated to a level outside its norm due to persistent external pressures, it will eventually sustain damage or long-lasting alterations in the related physiology [4]. 

Following chronic exposure to a potent addictive substance, the original response to the drug tends to diminish so that more, or more frequent, exposure is required to elicit the strength of the initial reaction. This process—the development of *tolerance*—is a cardinal characteristic of drug addiction. The neuro-adaptive changes in the brain's reward pathways that give rise to this phenomenon only occur when levels of reward stimulation by addictive drugs, or other dopamine agonists, become excessive. Specifically, the neuro-adaptation that occurs is a postsynaptic downregulation, which results in the reactivity of the reward pathway becoming desensitized [107]. For instance, reductions of striatal dopamine receptor levels, and of dopamine signaling strength, have been observed in response to repeated psychomotor stimulant administration [108–111]. There is also evidence of decreased brain cannabinoid receptor levels in human subjects who chronically smoke cannabis [112]. Consequently, as a result of the high and sustained levels of reward stimulation in substance abusers, and the subsequent downregulation, a pronounced decrease occurs in the individual's sensitivity or responsiveness to any kind of reward—a motivational and mood state known as *anhedonia*. 

Preclinical research has also demonstrated downregulation of dopamine receptor availability in rodents following chronic exposure to high fat foods [113], as well as markedly reduced basal levels of dopamine in the nucleus accumbens (assessed by microdialysis procedures) after 5-day access to a high-fat diet in obese-prone compared to obese-resistant animals [114]. Other evidence of downregulated striatal dopamine signaling has also been found in some groups of adults with obesity. For example, using positron emission tomography (PET), a group of morbidly obese adults were found to have significantly lower D2 receptor densities in the striatum compared to a group of nonobese controls [115]. These findings were replicated in more recent study of obese women and nonobese controls using SPECT imaging [116]. There are, however, important caveats to consider when drawing conclusions from these particular results.

Neuroimaging studies simply illustrate the current state of brain-circuitry activation, and they have mostly used case-control designs in addiction-risk research, constraining the ability to separate *causal* factors from *consequences* of the addictive behavior [117]. Self-report measures of reward sensitivity are also limited by the incapacity to distinguish antecedents from outcomes in those with addiction disorders. In other words, the evidence indicating a dampened down dopamine signal in some cases of obesity could simply be a reflection of the neuro-adaptation to excessive brain stimulation from potent dopamine activators like foods high in sugar and fat. By contrast, the study of genetic variation underlying addictions is able to address causality and is grounded on the premise that exposure to various environmental influences—in combination with one's inherent biology—determines the initial response to drugs as well as the neuro-adaptations that contribute to the transition from casual use to the addicted state [118]. 

#### 2.3.2. Escalation of Intake and Binge Consumption

While there is debate about precise mechanisms, another hallmark of addiction is that extended access to, and use of, drugs can precipitate a rapid escalation of intake, often in the form of binge-like consumption [119–122]. A similar response has been seen in animals exposed to high fat/sugar diets [93]. Based on sound theory and good empirical evidence, Alsio and colleagues [123] have proposed a “feed-forward” system involving neural adaptations in both the regulation of homeostatic and of hedonic hunger to explain the development of this behavioural pattern. Specifically, diets that are persistently high in fat and sugar promote a positive feedback signal that is experienced as an elevated or sensitized preference for, and consumption of, palatable foods, which in turn contributes to increased tendencies to overeat. The result can be a vicious cycle of craving and compulsive overeating, which is relatively resistant to change and highly prone to relapse after attempts to restrict palatable food intake. Such a pattern is clearly very similar to the psycho-behavioural blueprint that defines drug addiction. 

**Figure 1:**
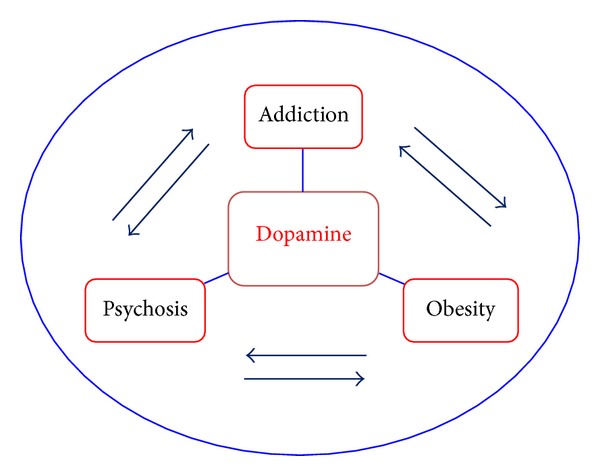
Comorbid interconnections among psychotic, addictive, and compulsive disorders and their biological links to dopamine regulatory brain pathways.

Not only does dopamine play a pivotal role in the compulsive nature of addiction disorders and overeating, but it is strongly associated with the pathophysiology of *psychosis*, all of which are themselves interconnected [124], as depicted graphically in Figure 1. Evidence for the associated network among these conditions comes not only from clinical practice but also from a growing body of research findings. For example, some Parkinson's patients treated with L-dopa experience episodes of drug-induced psychosis, especially when the drug is given at high doses [125, 126]. This is very similar to the dose-dependent, transient psychotic/paranoid symptoms associated with cocaine, cannabis, and methamphetamine abuse [127–129]. Compulsive behaviours such as excessive spending/shopping, binge eating, and hypersexuality can also be provoked by longstanding exposure to dopamine replacement therapy in Parkinson's patients [130, 131].

Considered from the “flip-side” perspective, the neuroleptic drugs used to treat psychosis dampen down dopamine signaling and, as a consequence, tend to foster symptoms of Parkinsonism, as well as obesity, in some vulnerable individuals [132, 133]. There is also a close relationship between psychotic disorders and a host of addictions such as to cigarette smoking, alcoholism, and opiate abuse [134–136]. And finally, there is substantial evidence that obese individuals are more likely to engage in a variety of addictive behaviours compared to their lean counterparts [137]. For instance, in a sample of almost 200 adults, we have recently found that those with obesity were significantly more likely to smoke cigarettes (*p* = 0.026), to engage in excessive spending (*p* < 0.0001), and to have episodes of binge eating (*p* < 0.0001) compared to their normal weight counterparts [138]. 

## 3. A Dimensional View of Overeating 

Most overweight and obese individuals do not display any distinctive, or especially aberrant, patterns of food consumption, nor do they typically suffer from clinically significant psychopathology or other behavioural disturbances. Some individuals overeat because the supreme self-regulation required to exercise dietary restraint is very difficult to muster in our obesogenic food environment [139]. Others may overeat because they are highly sensitivity or responsive to rewarding stimuli, of which palatable food is a primary target [140, 141]. Still others may use food to “self-medicate” a negative affect because sweet tastes have well-known mood-elevating, and pain-suppressing, properties [142]. However, a smaller proportion of individuals do show signs of disordered or dysregulated overeating, in varying degrees of severity, which are typically characterized by episodes of compulsive intake and/or nocturnal hyperphasia, and are associated with a range of psychiatric co-morbidities like depression, attention deficit/hyperactivity disorder (ADHD), and psychosis [143]. Despite some earlier suggestions to the contrary [144] obesity itself is not a mental disorder. There may, however, be some individuals, whose overeating and weight gain are caused by a psychiatric disturbance like depression [145], and others where the cause relates to their treatment, as seen in the case of neuroleptic-induced weight gain [124]. 

**Figure 2:**
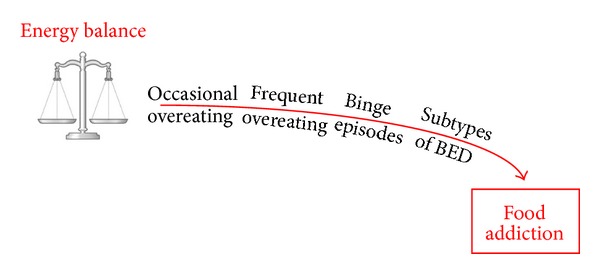
A downwardly escalating dimension of (over)eating and behaviours reflecting increased severity and compulsiveness.

In the following sections of this review, the primary aim is to consider theoretical arguments and related evidence for the proposed view that *overeating* is a dimensional prototype reflecting collectively its severity, its degree of compulsiveness, and its clinically significant level of personal impairment, as presented in Figure 2. It will also be argued that chronic overeating—irrespective of the motivational factors that may have prompted this behaviour—increases the risk that over time food consumption will become more uncontrollable and intractable to change. Eventually, and in certain susceptible individuals, the nature of the behaviour will closely resemble a drug-addiction disorder [146]. 

At the beginning of the “eating continuum” is what we might call “homeostatic eating” reflected largely by a stable body weight falling within the “healthy range” (BMI between 18.5 and 25) according to regulatory institutions like the World Health Organization [147]. This state does not reflect any particular daily calorie intake, but rather that the energy consumed matches the energy expended in the form of physical activity and one's metabolic requirements. Further along the gamut we have “passive overeating”, but with little or no concomitant psychopathology. The majority of individuals whose weight increases slowly over time, are largely unaware of living in a chronic state of positive energy balance. We live in an environment where it is enormously difficult for even the most restrained individuals to eat a diet sufficiently reduced in calories to match their energy expenditure. The proliferation of high-calorie fast food outlets and the convenience and low cost they provide, the increased portion sizes served in grocery stores and restaurants, the vast array and constant availability of food, and the enhanced taste experience of eating due to the sweeteners added to many foods and beverages, have created a collective inertia that is difficult to escape. 

Still further along the eating continuum we see mild and intermittent “loss-of-control” eating, which manifests as episodic binges that tend to become more compulsive and more frequent in some individuals over time. When these behaviours become severe—as reflected by the downward curve displayed in Figure 2—a diagnosis of binge eating disorder (BED) may be warranted. Like virtually all psychiatric disturbances, despite a uniform clinical label, there is considerable heterogeneity in symptomatology, aetiology, and outcome in BED. Its heritability is also well established, with estimates suggesting that the genetic contribution is about 50%, and that its prevalence is significantly greater in women [148–150]. However, the quest for specific risk-markers for this disorder has been obstructed considerably by the complexity of the phenotype. 

Although BED research is still relatively limited compared to more established disorders, it is important to identify and understand subtypes of BED with specific psychobiological profiles that may convey different vulnerabilities to environmental risk factors and therefore indicate different targets for intervention. Such an approach will better inform personalized treatment strategies for those who struggle with overeating and weight gain. For example, some individuals with BED have a BMI in the normal healthy range, and in many cases, are difficult to distinguish from normal-weight adults with nonpurging bulimia nervosa. 

Among overweight adults with BED—a group who comprise the large majority of individuals with this disorder—some manifest virtually no psychopathological or behaviourally-aberrant differences from overweight adults without BED *except* for their binge eating and their elevated responsiveness to highly palatable food. Others, however, express significantly more severe and compulsive symptomatology and more psychopathological characteristics. They also display a clinical profile strongly akin to individuals with conventional drug-addiction disorders. In these individuals it seems eminently more appropriate to describe their condition as a “food addiction” and to recognize the biological state of dependence and the downwardly spiraling *pathophysiology *propelling the behaviours, rather than viewing it as a condition where *psychopathological *factors are largely driving the chaotic episodes of loss-of-control eating. 

To recapitulate briefly, near the low end of the (over) eating continuum are those who—despite excessive calorie intake and weight gain—display little or no behavioural pathology and psychiatric disturbances, followed by those with a similar lack of pathology except in relation to their compulsive binge eating. At the higher end of the continuum are those with BED who display significant psychopathology and strong addictive tendencies towards food—indeed meeting the diagnostic criteria proposed for “food addiction” [151] based on the DSM-IV criteria for substance dependence. In particular, these individuals are highly impulsive, manifest significant mood disturbances, and display emotionally-driven bouts of overeating. 

The following sections will provide a psychobiological discussion of BED followed by a detailed discussion of the evidence supporting the concept of food addiction. Arguments will then be presented, with supporting research evidence, that food addiction is largely a severe and compulsive form of BED rather than a clinically distinct condition with a unique aetiology and clinical course.

### 3.1. Bingeing and Binge Eating Disorder (BED)

BED is an overeating syndrome seen much more frequently in those who are substantially overweight [152–154]. Frequent bingeing can also occur episodically in those who do not meet the criteria for an eating disorder according to the DSM [155]. The cardinal features of BED are recurrent binge eating without compensatory weight control behaviour such as self-induced vomiting, diuretic use, and excessive levels of physical activity (DSM-IV). The disorder is also associated with eating large amounts of food in the absence of hunger, eating alone because one is embarrassed, and feeling guilty, depressed, and disgusted after the binge episode. 

BED also appears to be a behaviourally distinct subtype of obesity with an unique risk profile reflecting a heightened response to rewarding stimuli. In particular, individuals with this disorder display a hyperreactivity to the hedonic properties of food as seen by greater food cravings, preoccupation with thoughts of food, emotionally-induced overeating, and a greater preference for sweet and fatty foods [102, 156]. Moreover, it is no longer seen as a syndrome exclusive to adulthood as it once was. In recent years, bingeing or “loss-of-control” eating has also become prevalent in middle childhood and early adolescence [157]. Also pertinent to the theme of the current review is the observation that the 12-month prevalence of binge eating and illicit drug dependence occurs at roughly the same rate—in about 2% to 3% of the population [158].

#### 3.1.1. Animal Studies of Binge Eating

Using animal paradigms to model psychological disorders is challenging and inevitably cannot represent the complexities seen in the human condition, nor the intricate factors that give rise to their development. On the other hand, animal models provide the opportunity to manipulate conditions, induce behavioural or physiological changes, and thereby isolate important causal factors that would be impossible to do in human research where studies are typically carried out in those with pre-existing conditions. 

Although several animal models of binge eating have been developed in recent years, the *limited access* paradigm is one of the most rigorously tested and is based on relevant DSM-IV criteria for binge eating [158]. In the typical protocol, one group of rats is given limited and intermittent access to fatty and/or sugary foods 3 times a week while the control group has access to the same foods every day of the week. Neither group is food deprived. Over a period of several weeks, the “intermittent” group shows increased intake of the palatable food at a significantly greater degree than the daily access group. In a brief period of time, they also eat larger amounts of the palatable food than is considered normal [159]. 

Similar findings have been observed in nonhuman primates who were given limited access to chocolate candy [160]. While excessive eating was clearly based on the palatability of the food in this paradigm, it was also moderated by social factors since the submissive animals—both male and female—ate more palatable food than their dominant counterparts [160, 161]. Interestingly these findings mesh with evidence that submissive primates also self-administered more cocaine than their dominant counterparts [162]. In summary, the *limited access* model has good clinical validity because many people who binge eat try to limit their access to sweet and fatty foods, and then typically binge on the very foods they have unsuccessfully tried to restrict [163, 164].

Another theory of binge eating with good face validity is the *maternal separation* animal model, which implicates stressful experiences occurring in childhood with behavioural abnormalities later in life [165]. Specifically, neonatal maternal separation or isolation is associated with the development of binge-related eating if the animal is confronted with social or metabolic stressors later in life. Many studies have demonstrated that traumatic events early in life form a major risk factor for developing subsequent depressive disorders [166, 167]. We also know that individuals with compulsive, binge-like overeating frequently have cooccurring symptoms of depression and anxiety that predate the eating disturbances [168, 169].

#### 3.1.2. Biological Underpinnings of Binge Eating

The research has consistently demonstrated that repeated consumption of highly palatable food can produce dopamine signaling changes in the brain, which promote an escalation of intake [170]. It also appears that intermittently restricted access to highly palatable foods results in sustained stimulation of the reward system, which in turn fosters chronic overeating and binge-eating susceptibility [171]. In this regard, it is relevant that individuals with BED tend to under-eat during the mornings [172], but show a propensity for high-calorie nocturnal eating [173], and do greater snacking in the evenings [174] compared to weight-matched adults who do not binge eat. 

Neuro-scientific evidence of high reward responsiveness in BED is seen by the greater medial orbitofrontal cortex reactivity to food cues in people with this disorder relative to comparison groups [175]—a finding which accords with the important role of this brain area in drug cravings, and which confirms the significance of dopaminergic mechanisms in the aetiology of BED [176, 177]. Larger brain-activation differences in the amygdala and ventral striatum have also been found in obese adults with BED compared to weight-matched controls, suggesting a greater motivational sensitivity to, and visual processing of, palatable food cues in the former group [178]. The finding that obese binge eaters have greater increases in extracellular dopamine levels in the caudate nucleus, when presented with tasty food compared to nonbinge eating controls, provides further evidence of a *hyper-responsiveness* to reward in BED [179]. Additionally, a recent study has shown that exposure to personally relevant, high-calorie food cues elicited hyper-responsive fMRI BOLD reactions in overweight adults with binge-eating behaviour, and importantly, that the magnitude of these responses increased as the binge-eating symptoms increased in frequency [180]. Finally, and again in response to food cues, binge eating women showed hyper-responsiveness and increased neural communication between the ventral striatum and a widespread network involved in reward and action planning compared to weight-matched controls [181].

In accord with the evidence described above—that BED reflects a heightened responsiveness to reward—we recently reported that genetic markers reflecting enhanced dopaminergic signaling were positively related to the food-related sub-phenotypes of BED such as binge eating, food cravings, and emotional eating [94]. In addition, a multilocus genetic profile score—indicative of stronger dopamine responsiveness imparted by cumulative functional genotypic influences [182]—was significantly higher in a group of obese adults with BED compared to their age- and BMI-matched counterparts [101].

#### 3.1.3. Emotional Dysregulation in Binge Eating

In clinical practice, stress, depressed mood, anger, and irritability are the most commonly reported triggers of binge eating [183]. Negative “everyday” emotions are also more prominent in those with BED compared to obese control participants [184, 185]. Such findings have prompted efforts to better understand the possible causal mechanisms for these associations. An important target is the role of stress responsiveness as a moderating factor in the development of binge eating. For instance, obese patients with BED appear to have significantly higher than expected co-morbid rates of posttraumatic stress disorder (PTSD) than weight-matched controls—a relationship that was also characterized by eating-related psychopathology [186]. Other related research has demonstrated a greater cortisol response to stress in this group compared to obese controls [183]. 

These findings have also been corroborated by experimental stress-induction laboratory studies. In one such paradigm, obese BED participants in the stress condition demonstrated a stronger motivation to eat and a weaker satiety perception compared to those without BED and who were not in the stress condition, suggesting that BED is more affected by stress than is seen in weight-matched controls [187]. There is other evidence of increased cardiovascular activity, and a greater sadness and insecurity response to stress induction in BED participants compared to their controls [188].

Preclinical evidence also supports this conclusion. Socially-isolated female rats (single-caged housing is an established stressor in these animals) showed a greater intake of cookies (when they had access to this food) compared to their standard chow, and they demonstrated more depression-like behaviours than the controls who were housed in group cages [189]. In addition, and not unexpectedly, basal levels of plasma corticosterone were elevated in the stressed animals. Previous research has clearly demonstrated that administration of glucocorticoids and corticosterone leads to hyperphasia and weight gain [183]. 

Findings such as those described above have generally been interpreted as an indication that the excessive ingestion of palatable food is largely a form of “self-medication” to restore or improve a disturbed affect. What has not been considered, however, is that the more sustained negative affect in those with BED could be a response to pronounced withdrawal symptoms as defined by the diagnostic criteria for drug addictions. Withdrawal symptoms include anhedonic mood, irritability, and a variety of somatic symptoms. The return to substances relieves this discomfort and therefore the behaviour is maintained through a negative reinforcement process whereby continued use avoids the unpleasant symptoms. Indeed, the same mood improvement is seen following a binge in those with BED [185]. It has also been found that negative mood tended to improve just prior to the binge [185]—a finding that is reminiscent of the cue-induced neural activation of striatal brain regions that occurs even before the drug is administered [190]. In other words, while the frequent eating of highly palatable foods may be initiated by a high sensitivity to rewarding stimuli, over time attempts to refrain from binge eating are followed by withdrawal symptoms in the form of depressed and irritable mood, which then restores the overeating to calm the distress. Like other addiction disorders, the cycle becomes increasingly compulsive and results in repeated failures to refrain from the behaviour. 

### 3.2. Food Addiction

While “behavioral addictions” have been recognized by scientists and clinicians for many years [191], they have only recently been endorsed by the American Psychiatric Association with the proposed classification of Addiction and Related Disorders in the forthcoming *Diagnostic and Statistical Manual[DSM]-5* [192]. The DSM diagnostic shift is timely in light of growing evidence that excessive consumption of hyper-palatable food is an identifiable clinical entity with striking biobehavioral parallels to drug abuse [60, 193, 194]. Indeed, as we have seen in Section 2.3 above, the concept of food addiction has recently generated a strong public interest as well as an increased research focus. 

#### 3.2.1. Animal Models of Food Addiction

The food-addiction construct earned its scientific credibility largely from evidence that some aspects of this syndrome can be very successfully modeled in animals. Rodent models of food addiction have typically used behavioral paradigms based on experimentally inspired analogues of the *Diagnostic and Statistical Manual *(DSM-IV-TR) [195] criteria for substance dependence. For example, “tolerance” has been operationalized as an increase in brain-stimulation reward (BSR) thresholds following extended exposure to addictive drugs [196]. The inference is that stronger excitation is required because the sensitivity of the organism has been de-sensitized by chronic drug use. When extrapolating this principle to eating behaviors, rats with 18–23 hours/day access to palatable energy-dense food displayed a worsening deficit in brain reward function, as seen by their progressively elevated BSR thresholds across days, compared to rats on a standard chow diet [196].

Escalation of intake over time has been used as a marker of the substance-dependence criterion “taking the substance in larger amounts than was intended” [197]. In this regard, there is reliable evidence that rats, fed an intermittent diet of sugar, develop a pattern of copious consumption resembling human cases of binge eating [93, 198]. These studies show that a sugar-enhanced diet increases daily food intake over time, and that following its removal, the animals show aggression, anxiety, teeth-chattering, and head-shaking—all symptoms associated with “withdrawal” from drugs like heroin. Similar increased intake has been found when animals were given high-fat [45] or other highly palatable diets [196]. Evidence of withdrawal has also been observed in animals showing depressive-like symptoms after access to highly palatable food for 7 weeks, as indicated by increased immobility in the forced-swim test and decreased intake in the sucrose consumption test [199]. Interestingly, some of the depressive symptoms were abolished in these animals when they were given renewed access to the palatable diet, suggesting that high-calorie food can relieve withdrawal-induced negative effect, as well as fostering its onset. Recent evidence also indicates that rises in extracellular acetylcholine and decreases in dopamine in the striatal area contribute to the aversive state which characterizes both drug and palatable food withdrawal [200].

It also seems that compulsive patterns of palatable food intake only require a short time to develop, and that rats rapidly display a lack of flexibility in their food choices by rejecting standard chow even when the greatly preferred rich “cafeteria diet” is only intermittently available [201]. Even very limited (10 min) daily access to high sugar/fat diets can cause binge-like eating patterns and excessive weight gain in rats. Interestingly, however, this occurs without the physical and emotional withdrawal responses seen in rats with access to sugar-rich diets [202]—a finding which adds to the growing evidence that only minimal withdrawal symptoms are associated with diets rich in fat, or a combination of sugar and fat, compared to the very pronounced withdrawal symptoms following removal from a high-sugar diet [203, 204]. Various explanations have been proposed for these differences including the greater hedonic intensity of sugar relative to fat [202], and the more pronounced opioid-activating potential of sugar [203]. In general, it seems that a high-fat diet is largely responsible for weight gain while sugar may be mainly responsible for producing addictive-like behaviour including explicit withdrawal symptoms [194]. 

Another diagnostic criterion for addiction is the unremitting continuation of the behaviour in the face of unpleasant consequences. There are several examples of such compulsive food seeking and food intake in animals. For instance, binge-prone female rats tend, on average, to tolerate significantly higher levels of foot shock for access to Oreo cookies than their binge-resistant counterparts, confirming the “continued use despite negative consequences” criteria for substance dependence [205]. Other evidence of abnormal motivation for sweet and fatty foods has been demonstrated in paradigms where rats with extended exposure to a highly palatable cafeteria diet continued to eat even when the food was laced with quinine to produce an unpleasant bitter taste [201]. Preliminary data also confirm that compulsive eating in response to a highly palatable diet emerges only in a subset of animals with a genetically determined susceptibility [206]. 

Finally, while numerous studies have found behavioral and consummatory cross-sensitization from one addictive drug to another [93]—and between drugs and responsiveness to stress [30]—there is now good evidence that sugar intake also cross-sensitizes to drugs of abuse, and *vice versa* [198, 207, 208]. Interestingly, however, a recent study has shown that uncertain reinforcement with a sweet solution produced an enhanced locomotor cross-sensitization response to amphetamine compared to animals exposed to a predictable sweet reinforcement [209]. In other words, gambling-like reinforcement promotes more pronounced cross-sensitization to psychomotor stimulant effects—supporting the evidence that pathological gambling and compulsive/binge-eating disorder are regulated by similar brain substrates. Given the strong taste-appeal preference of salty foods, it is also important to note that animals pretreated with cocaine show a stronger salt appetite, and that these two substances show reciprocal behavioural cross-sensitization [210]. 

In conclusion, in any model of addiction it is fundamentally important to recognize the great variability in proneness to its development [211]. Only a subgroup of animals and people who are exposed to drugs of abuse, or highly-palatable food, seem to develop full-blown symptoms of dependence and loss-of-control over their intake [212, 213]—an observation that has galvanized attention on identifying the important risk factors, and the biological mechanisms underlying vulnerability [214]. One emerging area of interest stems from evidence that early-life stressors, and various dietary differences that arise from these stressors, are influential in the susceptibility to compulsive intake of palatable foods [211]. 

When assessing animal paradigms that support the food-addiction construct, an important consideration is that a pattern of compulsive intake—behaviourally similar to what we call *binge eating* in the human condition—is a universal feature of all models [211, 215]. In other words, animals do not display symptoms of withdrawal, tolerance, or any of the other analogue criteria for substance abuse, without also showing binge-like consumption of the experimental food. Moreover, all animal models require the introduction of highly palatable foods—typically those that are sugary or fatty—and sometimes a combination of both. Pivotally, compulsive intake does not occur in response to tasteless foods that comprise the chow that is typically fed to laboratory animals. 

#### 3.2.2. Human Studies of Food Addiction

Despite the persuasive evidence of sugar/fat dependence in rodents, there are limited parallel findings in human research except for anecdotal reports and various qualitative clinical accounts [68, 151, 216, 217]. Recently, researchers at Yale University advanced the field with their development of a measure which operationalizes “food addiction” using the DSM-IV-TR diagnostic criteria for substance dependence [218]. Their preliminary findings suggested that the *Yale Food Addiction Scale *(YFAS) has high convergent validity with other measures of eating pathology, especially binge eating, and may therefore be a useful tool for identifying individuals with compulsive and addictive tendencies towards food. In a more recent YFAS validation study, the high symptom group demonstrated faster reaction times in response to food cues versus neutral cues and reported higher attentional impulsivity, compared to the low symptom group—similar to findings in those with other addiction disorders [219]. 

Our group published the first case-control study of food addiction in obese men and women using the YFAS diagnostic criteria for classification [218]. The results provided very good evidence that food addiction is a discernible clinical syndrome characterized by psychiatric co-morbidities and a psycho-behavioural profile with strong similarities to those found in conventional addiction disorders like alcoholism and drug abuse. For example, those who met the diagnostic criteria for YFAS food addiction had a significantly greater prevalence of BED, of severe depression, and reported more pronounced symptoms of ADHD compared to their obese counterparts. In addition, they reported more intense food cravings and greater emotional overeating. They were also more impulsive than the obese controls. 

#### 3.2.3. Neurobiological Evidence

The last point (above) is particularly relevant given that hypo-functioning of prefrontal cortical systems—cognitive processes, known broadly as “executive functions” and regulated largely by dopaminergic circuitry—may be a cardinal feature of addiction disorders, especially as it contributes to proneness to relapse and poor success in recovery [220]. Moreover, as addictive behaviours escalate there appear to be growing deficits in the individual's ability to exert “self-control” and to resist engaging in excessive behaviours [146]. In other words, decreased activity in the prefrontal cortex has been consistently associated with relatively high levels of impulsive behaviours, and these findings relate both to the abuse of drugs [221] as well as to an overindulgence in palatable foods [222, 223]. There is, however, some evidence that drug-related cues are more potent triggers of drug-seeking behaviour than food-related cues are to eating, although the differences appear to be more a matter of magnitude than of quality [224].

Low brain serotonin function has also been widely associated with impaired emotional regulation, impulsivity, and impulse control disorders [225]. Based on experimental evidence that tryptophan (a precursor amino acid for serotonin) depletion tends to foster high consumption of sweet/fatty foods, and that it can also trigger depressive episodes, some have argued that overeating of highly palatable foods is fundamentally a form of *self-medication* to ameliorate dysphoric mood [226]. In light of the significantly more severe levels of depression in those with food addiction, and their reported proneness to emotionally-driven eating, these interrelationships may well explain a downwardly spiraling cycle whereby a biological predisposition to negative mood (e.g., low serotonin availability) could foster a voracious appetite for sugary and fatty foods. In our highly obesogenic environment with its proliferation of processed foods, this hungriness can easily be satisfied, in turn causing down-regulated brain reward pathways and further contributing to dysphoria and its consequences on eating behaviours.

Currently there has only been one neuroimaging study designed to assess biological correlates of food addiction [227]. Reward circuitry activation in the caudate and amygdala, in response to the anticipated receipt of food, was positively associated with YFAS-symptom scores. This study was, however, rather limited by a small all-female sample, and because only two participants met the diagnostic criteria for food addiction. However, in a recent study—and the first to provide genetic support for the food-addiction construct—our findings meshed well with these neuroimaging data [228]. A multilocus genetic index score, which reflected the cumulative effect of several functional markers on striatal dopamine signaling strength, was significantly higher in a group of overweight adults with YFAS food addiction compared to their weight-, age- and gender-matched counterparts. This relationship was also mediated by greater food cravings, bingeing, and emotional eating. Together, these findings support the view that, like BED, food addiction is a high reward-responsive phenotype of obesity. They also suggest a reward-based process model whereby a biologically based vulnerability contributes to increased risk for overeating, which in turn tends to promote addictive tendencies towards highly palatable foods. 

### 3.3. Food Addiction as a Severe Form of Binge Eating Disorder

As we have seen, the available research—albeit still very limited as it pertains to the human condition—consistently endorses the view that food addiction is an identifiable clinical entity with many similarities to drug addictions. At the same time, however, these investigations, and related work, have implicitly raised the question of whether the YFAS-inspired definition of food addiction simply reflects a more severe sub-type of BED. This issue is especially germane given the clear overlap in the proposed diagnostic criteria for the two conditions. They also share substantial symptom overlap [218, 229], they have many co-morbidities in common [218], and they appear to share similar biological underpinnings [101, 228]. 

The studies by Davis et al. [218] and Gearhardt et al. [229] found that about 50% of obese adults diagnosed with YFAS food addiction also met criteria for BED. Moreover, in an earlier study [230], using structured telephone interviews in women diagnosed with BED, the investigators found that 92% of their sample met the DSM-IV-TR [195] criteria for substance dependence when the word “food” was substituted for “drug” in the interview questions. Clarifying whether the two conditions are sufficiently different in aetiology and clinical course to warrant classification as separate pathological phenotypes of overeating is an important next-step in eating disorder and addiction research. Resolution will also help guide personalized treatment strategies for those who struggle with intractable and excessive food consumption [91]. 

As an initial attempt to assess our continuum hypothesis, we examined group differences on several variables related to demographic characteristics (age and BMI), patterns of overeating, personality risk factors, and co-morbid clinical symptoms [91]. In the first set of analyses, we compared two equivalent groups of overweight men and women who met DSM-IV-TR criteria for BED: one *with* co-occurring YFAS-diagnosed food addiction and the other *without*. Results indicated that the two BED groups were equivalent in age and BMI. On almost all other variables, however, the differences were highly statistically significant. Those with food addiction were much more likely to overeat for emotional and cue-driven reasons, they had more severe binge eating and food cravings, and they were more responsive to the rewarding properties of food. This group also had more addictive personality traits, was more impulsive, and had greatly elevated symptoms of depression compared to their non-food addict BED counterparts. While the food-addict group also reported greater ADHD symptoms, both currently and in childhood, these differences only approached statistical significance. Collectively, these findings mesh well with a related study of food addiction in BED patients where the number of YFAS *symptoms* (scores ranging from 0–7) was shown to correlate positively with Beck depression ratings, poor self-esteem, and difficulties in emotion regulation, as well as with certain measures of eating pathology including frequency of binge episodes [229]. Similarly, in a recent study of weight-loss treatment-seeking adults, YFAS symptom scores related significantly to increased depression, emotional eating, and binge eating [231]. In this study, increased symptomatology was also related to less weight loss after several weeks of treatment, suggesting that food addiction, with related signs of tolerance and withdrawal, may undermine efforts to lose weight in those trying to adopt better eating habits.

In the second analysis we carried out to test the continuum hypothesis [91], we compared the BED group *without* food addiction to a weight- and age-matched group of adults *without* BED and *without* food addiction on the same variables described in the first set of analyses. By contrast to the earlier comparisons, these two groups were very similar to each other and demonstrated only a few significant differences. For instance, and hardly surprisingly, the BED group had higher binge-eating scores and reported more hedonic eating and elevated food cravings than the non-BED controls. 

Despite the novel and interesting findings from our preliminary investigation, it is important to acknowledge that the results are based on a relatively small sample of participants with BED. Nevertheless, the highly statistically significant findings from the first set of analyses suggest that the sample was not under-powered since the mean differences were sufficiently large, relative to their standard errors, to demonstrate strong effect sizes. Unfortunately, however, in the total sample of mainly overweight adults, there were only a few individuals without a diagnosis either of BED or of food addiction, precluding a full factorial 2-way analysis of the data. Unfortunately such a skewed distribution is liable to be a problem for all future comparative research in this area because of the well-established associations between psychopathology and BMI in overeating disorders.

Although *normal-weight* adults only account for a small proportion of those with BED, they appear to be another distinct subset of the BED population, suggesting that body weight is also an important feature or correlate of the overeating spectrum. For instance, some studies have found that normal-weight BED adults are younger [232, 233], and that their binge episodes are less frequent [233] and of shorter duration than their overweight/obese counterparts [232]. Particularly relevant are the findings that those with normal-weight BED report a greater “drive for thinness” [234], more pronounced cognitive and dietary restraint [235], and significantly greater and more extreme compensatory weight-control behaviors like skipping meals, exercising excessively, and restricting their food intake [232]. 

Not surprisingly, a diagnosis of BED has been shown to predict weight gain over time [236, 237]. Indeed it is physiologically self-evident that chronic episodes of binge eating in the absence of any compensation for the excessive ingestion of energy substantially increase the risk for obesity as individuals grow older. Therefore—and because one of the diagnostic criteria for BED is the absence of “inappropriate compensatory behaviors like purging, fasting, and excessive exercise”—the boundary between normal-weight BED and nonpurging bulimia nervosa is clearly blurred, and their differences seem to be largely a matter of calorie-sparing severity. 

## 4. Summary and Conclusions

In the upcoming May 2013 release of DSM-5, there will be a number of notable changes to the status of BED. First, it will move from the Appendix of the previous DSM and be included as a free-standing disorder in the new version of this manual. The criteria for diagnosis have also been relaxed in that the required duration of symptoms has reduced from 6 months to 3 months, and the frequency criterion for binge episodes has dropped from at least twice a week to at least once a week, on average [238]. Together these changes should capture a greater number of individuals suffering from a clinically significant impairment in their compulsive overeating. It has been estimated, however, that while the point prevalence of BED will naturally increase with the more liberal criteria, the lifetime prevalence is unlikely to increase markedly [239]. Another important change in DSM-5 is the addition of severity ratings based on the number of weekly binge episodes—an index also of the compulsiveness of the behaviour. 

It is anticipated that the inclusion of more individuals with debilitating BED-like symptoms, without compromising the validity of the BED diagnosis, will allow future researchers to assess the hypothesis presented in this paper, that “food addiction” is not a separable entity from BED but rather that it reflects—at least in most case—a more severe and more compulsive subtype of BED. In proposing a continuum hypothesis of overeating it is also important to acknowledge that at the extreme of some dimensions, a sort of “chaos” ensues rather than an orderly progression of the underlying or defining characteristics in degrees of magnitude. In other words, there may be a qualitative difference as well as a quantitative difference in the clinical picture at the high (food addiction) end of the overeating continuum such as can be seen, for example, in the case of Obsessive Compulsive Disorder. In that condition, the obsessive-compulsive personality traits not only manifest as severe, rigid, and debilitating characteristics, but there is the emergence of associated cognitions and behaviours that are chaotic, aberrant, and often discernibly unrelated to features of the personality. 

In conclusion, as we have seen in this review, an increasing number of scientists and clinicians subscribe to the view that compulsive overeating—a quintessentially defining feature of BED—shares pronounced similarities with conventional addictions like substance abuse [60, 211, 240, 241]. Not surprisingly, such discussions have inspired cautious conclusions that *some* forms of excessive overeating are most appropriately viewed as an addiction disorder. While evidence for the validity of the food-addiction construct is rapidly gaining ground, it is inevitable that this perspective has also found its detractors [242]. Unfortunately, some of those participating on the negative side of the debate have conflated obesity and overeating with “food addiction,” in presenting their arguments—an issue which has clouded the possible distinctiveness of the concept and muddled efforts to properly assess its diagnostic utility. 

It is clearly a misinterpretation of the research for anyone to perceive that supporters are claiming that *all* cases of overeating—even prodigious overeating—reflect an “addiction to food,” in the same way as it would be illogical to maintain that all heavy drinkers are alcoholics. In other words, there are no explicit assertions from advocates of the food-addiction concept that more than a subset of the population could, or would, be affected by this condition. Like virtually all psychiatric disorders, including drug abuse and behavioral addictions such as pathological gambling, there is a relatively low base rate in the population, even though the related symptoms and endo-phenotypes exist in the general population along a continuum of varying magnitude or severity. Most individuals who use addictive substances—drugs or highly palatable food—do not develop dependence. Many contributing elements explain an individual's propensity to these disorders. Moreover, it is highly unlikely that a homogeneous set of risk factors pertains to all phases of their development [243].
